# Why Uncertainty Is Essential for Consciousness: Local Prospect Theory vs. Predictive Processing

**DOI:** 10.3390/e27020140

**Published:** 2025-01-28

**Authors:** Francis Heylighen, Shima Beigi

**Affiliations:** Center Leo Apostel, Vrije Universiteit Brussel, 1050 Brussels, Belgium

**Keywords:** consciousness, predictive processing, global workspace, entropy measures, uncertainty

## Abstract

We present and develop local prospect theory (LPT), a novel framework for understanding consciousness, and, in particular, subjective experience and free will. While predictive processing (PP) theories model the brain as trying to optimize the accuracy of predictions, LPT sees uncertainty as an essential feature of conscious decision-making. This is achieved by creating a “local prospect”—a range of potential developments colored by subjective experience from which an agent can freely choose how to react. Drawing on global workspace theory, LPT conceptualizes consciousness as a self-maintaining process of circulating neural activation, creating a temporary working memory where thoughts and feelings coming from different brain modules enter into an asynchronous, non-linear interaction. This contrasts with unconscious processes, which operate automatically and deterministically. LPT proposes entropy-based measures, including the determination of actions by conditions and the breadth of prospect, to quantify the range of potential developments considered. This framework allows us to understand Buddhist practices and concepts, such as mindfulness, liberation from attachments, and meditation, which broaden consciousness and de-automatize reactions by reducing the influence of conditioning. The proposed prospect measure may be operationalized by indicators such as the variety of action, breadth of perception, and unpredictability of behavior, thus allowing for the empirical testing of the theory.

## 1. Introduction

*“Between stimulus and response there is a space. In that space is our power to choose our response. In our response lies our growth and our freedom.*”

(quote attributed to Viktor Frankl)

We have recently proposed a new approach for tackling the notorious “hard problem of consciousness” [1,2]. This problem consists of trying to understand the nature and function of *subjective experience*, which is also known as *phenomenal consciousness* or *qualia*. Our approach, which we have named the local prospect theory (LPT) [3,4], is inspired in part by existing neural models of consciousness, in particular the global (neuronal) workspace [5,6] and the adaptive resonance theory [7,8], and by Buddhist ideas about freedom from conditioning [9].

Over the past two decades, another fundamental theory of cognitive processing in the brain has been elaborated in extensive detail: predictive processing (PP) [10,11,12,13]. PP has made a deep impact on a wide range of domains—from neurophysiology across psychology to AI and philosophy—by clarifying the mutual dependence between bottom-up information processing (perception) and top-down interpretation and inference (prediction). While PP is not a theory of consciousness as such, several authors have suggested that it may help us to solve the hard problem of consciousness [14,15,16].

Like PP, our novel local prospect theory recognizes the fundamental role of the brain in predicting or anticipating perceptions, conceptions, and actions. In that sense, it can be seen as extending or building on PP’s insights. However, there is a fundamental difference between LPT and PP, which for us defines the essence of consciousness. Predictive processing, like other mathematical models inspired by physics, describes the functioning of the brain as an optimization process: the brain is trying to make *the best possible* prediction. That means that its processes strive to minimize uncertainty—as formalized by the information-theoretic measures of surprisal or free energy [17]. In our LPT analysis, on the other hand, consciousness is characterized by a free choice from a range of options, implying an essential uncertainty about what comes next. From this perspective, an optimization model like PP, which selects one right solution (interpretation or action), appears like a good model of unconscious processing. However, it lacks the aspect of consideration, deliberation, or “free will” that is characteristic of conscious decision-making and of our ability to go beyond conditioned responses. This freedom entails a range of possibilities from which a choice is to be made. That range is what we call a *local prospect*. It can be modeled as a probability distribution with a non-zero entropy over a space of potential actions.

In the following, we will summarize the local prospect theory and review its similarities and differences with PP and its associated “active inference” framework. We will discuss several examples to demonstrate how LPT answers the hard problem of consciousness and the question of free will, in a manner that PP and related theories fail to do. We will further argue that LPT explains and corroborates a number of observations and techniques to control consciousness that were developed in Buddhism. We will finally suggest methods to operationalize LPT, in particular by developing mathematical and empirical measures for the “breadth of awareness” that LPT assumes.

## 2. Sense-Making in LPT and PP

In LPT, the most fundamental function of cognition, and therefore of the brain, is to *make sense* of the situation as experienced by an individual agent [18,19,20]. Sense-making means understanding what is going on, anticipating what may happen, and thus being prepared to intelligently choose actions appropriate for the situation. That is necessary because an agent, as a biological organism that has evolved to be good at survival, must be able to deal with any challenges it is likely to encounter. These include problems to be solved or dangers to be evaded as well as opportunities to be exploited.

Making sense of a situation and tackling the challenges inherent in that situation is a process that can be decomposed into the following aspects:

1. **Perception**: acquiring relevant information about the situation by means of the sensory organs, such as eyes and ears;
2. **Identification**: recognizing specific categories of phenomena (e.g., a cat, a table, or a lightning bolt) with known properties in these perceptual data;
3. **Explanation**: inferring a reason, origin, or cause for the situation that was perceived, thus coming to understand why these phenomena are present;
4. **Prediction**: using knowledge about the nature, properties, and origin of the identified phenomena in order to anticipate what may happen next;
5. **Intention**: evaluating the different perceived, conceived, and anticipated phenomena as better or worse, thus mapping out desirable outcomes (goals, intentions) as well as undesirable ones (dangers, problems);
6. **Action**: selecting and performing actions likely to achieve the intentions and to evade the dangers, thus improving the situation for the individual insofar as this is possible.

These six components characterize the interaction between an intelligent, goal-directed agent and its environment. They were inspired both by a cybernetic analysis of goal-directed action [21,22], and by the concept of a *worldview* as a broad scheme for making sense of the world in which one lives [23,24].

The “active inference” interpretation of predictive processing (PP), which also drew inspiration from cybernetics [25], assumes roughly equivalent functions of brain activity, albeit from a different perspective. The standard PP account sees the basic functions of the brain as not only prediction (component 4), but as inferring the likely identity, source, or origin of perceptions (components 1, 2, and 3), by using Bayesian reasoning to calculate the probabilities of different phenomena causing the perception, and then inferring the explanation with the highest probability. This explanation is used to predict further perceptions which are then compared with the perceptual data as they come in. When perception and prediction do not match, an error signal is generated that will trigger an inference process to find an explanation that produces a more accurate prediction. In this way, the brain seeks to maximize the accuracy of its predictions, while minimizing surprises (perceptions that were not predicted).

The *active inference* extension to PP rather counterintuitively postulates that intentions (component 5) and actions (component 6) are also predictions [26,27,28]. For example, when an organism is hungry, the extended PP account does not state that the organism has the *desire* or *intention* to eat, but that its brain *predicts* that it will eat. As long as there is no food, this prediction is in error, and therefore the brain will try to infer a better “explanation”. This is a so-called *active* inference, i.e., an inference that takes the form of an action in the real world, where that action is supposed to eliminate the error, by actually obtaining the food that the brain has “predicted” would be eaten. Thus, in the extended PP account, actions are merely externalized versions of the predictive inference processes inside the brain.

To us, this seems like an unnecessarily contrived formulation of goal-directed action, with the only function of reducing everything to predictions. We agree that there is a prediction aspect to action, in the sense that the brain plans or expects that the selected action will bring the situation closer to the intended goal. However, the goal itself is not predicted, but chosen among an extremely wide range of possible developments that are more or less desirable according to the organism’s value system. As a philosopher noted, the railway timetable allows you to predict which train will reach which city at which time. However, it does not tell you which city you intend to travel to: that depends on your personal preferences [29]. PP tries to circumvent this problem by postulating that our instinctive preferences (such as avoiding hunger) function as predictions that are hardwired into our brain, because the organism has been selected for survival. However, there is such a great uncertainty about how these hardwired preferences would be realized—if ever—that calling them “predictions” seems more like an abuse of language than like a clarification of the underlying mechanism.

Making abstraction of the terminology used, the active inference account and the local prospect theory agree that the six components listed above are essential aspects of the way the brain makes sense of a situation. The difference is that LPT sees the elementary processing steps underlying each aspect not as predictions, but rather as what is known in AI as *condition–action rules* [30,31]. These have the form: **if** condition *X* holds, **then** perform action *Y*. The short notation is: *condition* → *action* or *X* → *Y*. Predictions can then be seen as a special case of condition–action rules, with the action consisting of inferring an additional condition that is predicted to follow *X*. Such a rule could be noted as: *condition 1* → *condition 2*. This predicted *condition 2* can then function as input for another condition–action or condition–condition rule.

From a cybernetic perspective, an agent can only achieve its goal of survival by effectively *acting* in the world so as to reduce the deviation between its present condition and its preferred condition or goal. Prediction is then merely a more recently evolved mechanism supporting the selection of adequate actions. It takes the form of an internalized action (inference) that helps the organism to anticipate and plan developments in the outside world [20]. Goals can then be seen as implicit attractors of the dynamics of condition–action rules, i.e., final conditions to which a sequence of actions tends to converge [22]. From now on, we will therefore consider mental processes initiated by the agent (such as inferences, predictions, or decisions) together with its physical activities as all being *actions*.

## 3. Prospect vs. Prediction

This difference between LPT and PP—where LPT sees inferences as internalized actions, while PP sees actions as externalized inferences—is essentially one of formulation or point of view. In that respect, LPT and PP appear like complementary perspectives that are compatible in their understanding of fundamental cognitive processes. The more important difference is the role of uncertainty, which leads us to define the notion of *prospect*. The difference between prediction and prospect is that a prediction singles out a particular development as the one that *will* occur. In our theory, we define a prospect as a range of developments that *may* occur. Some of those are more probable or desirable than others, but, in general, none is certain. The word “prospect” in English has two common meanings: (1) the envisaged possibility, likelihood, or chance of some future development; (2) an outlook across a landscape. Both are special cases of our more general notion of prospect. The landscape interpretation will help us to explain the role of uncertainty and suggest a way to build a mathematical model of prospect.

The prospect over the landscape in front of you is what guides you in deciding where to go, i.e., which path to follow, which landmark to choose as your next destination, and which obstacles or difficult terrain to avoid. Hiking across a landscape is a concrete example of the way an agent interacts with its environment, continuously deciding what to do next based on its understanding of the perceived situation. That understanding or prospect includes immediate obstacles and affordances, such as the muddy puddle right in front of you and its relatively dry sides left and right that you could step on. It also includes more remote potential destinations, such as the mountain hut in the east or the wood in the south that you see far way. While trekking through open terrain, you must constantly make both immediate and more long-term decisions about where to go next, depending on the conditions as you perceive, understand, and predict them.

The resulting course of action, navigating efficiently across a landscape of opportunities and dangers, is common to all goal-directed agents [32]. We even find it in the directed movement of bacteria known as *chemotaxis*, during which the bacterium samples the local concentration of chemicals, such as food and poisons, and uses that perception to steer towards the food and away from the poison [22,33]. Our concept of prospect extends and generalizes this idea from concrete, physical landscapes to abstract, conceptual landscapes of potential developments. In mathematical terms, this means that we generalize from ordinary, three-dimensional space to a multidimensional state space defined by agent-relevant variables, and from a geological landscape of hills and valleys to a fitness landscape, where attractive destinations are characterized by high fitness and obstacles or dangers by low fitness.

Nevertheless, a fitness landscape is not yet sufficient to capture our notion of prospect. Traditional models of search on a fitness landscape assume that the agent has only a local knowledge of the fitness of a state: it can only evaluate the immediately neighboring states, lacking any knowledge of the path that leads to the global maximum of the fitness function. In an actual landscape, you typically also cannot see the optimal path that leads to the most desirable destination, because there are too many obstructions and the fuzziness of distance that hinder your view. However, you generally can see farther than the immediate neighborhood, although the accuracy of your prospect decreases with the distance [32]. That is what we mean by a *local prospect*: you do have some degree of foresight of where to go next, but only at a short range; you have no global, “God’s eye” overview of the state space.

That implies that the prospect is inherently *uncertain*: you have limited information to guide you in your decisions. But while you go forward you acquire additional information as places behind obstacles become visible or vague impressions are resolved into sharper detail. At the same time, the places you left behind disappear from your field of vision. Thus, your movement through the landscape is accompanied by a continuously changing prospect, where some things come temporarily into sharp focus while others remain or become to different degrees vague and uncertain [32].

The same shifting prospect accompanies cognitive processes in general. The task of the agent is to choose a good path through this patchily visible landscape of possibilities, which are to various degrees predictable or uncertain. The simplest strategy is *local optimization*: ignore all the faraway, uncertain prospects, and consider only the immediately available next steps. From these, choose the apparently best one. If it turns out to be a bad choice, the resulting error signal tells you to try the next best option. If this is still not satisfactory, choose the next one, and so on. This is the strategy followed by bacteria, who anyway can only sense the local concentration of chemicals. It is also the strategy assumed in PP models of cognition. These models function well in describing automatic processes of perception, understanding, and prediction in the brain. However, this is not the strategy followed in deliberate, conscious reflection. To explain this, we need to investigate the difference between conscious and unconscious activities.

## 4. Conscious vs. Unconscious Processes

The great majority of cognitive processes in the brain happen outside of consciousness [34,35,36]. We may be conscious of the result, but we are typically not conscious of how that result was achieved. For example, identifying an animal, such as a cat, is something we do automatically without having any notion of the complex stages of processing that lead from the scattered light stimuli on the retina to the conclusion that this is a cat. It is only recently that complex AI programs based on deep learning neural networks have been able to replicate that seemingly trivial feat [37,38]. Another example of an apparently easy activity is walking on two legs while maintaining balance. Again, it has taken AI researchers decades to develop software that can make a robot walk the way a human does, albeit still clumsily [39]. A last example of a complex cognitive process that we perform automatically and nearly effortlessly is understanding the words of a sentence spoken against a noisy background—something AI programs still struggle with.

The PP approach with its models of Bayesian inference clarifies how the brain selects the most likely interpretation of such initially ambiguous visual or auditory stimuli. Its active inference extension helps us to understand how the brain guides the movement of the muscles in our legs and rump in order to plan and execute the next step while walking. What characterizes these kinds of processes is that the brain needs to select *the one correct solution*. The animal you saw is either a cat or it is not. The step you take either makes you move forward, or it makes you stumble. If the solution inferred turns out to be wrong, the PP error-correcting mechanism will automatically select the next best one.

Conscious decision-making functions in a different way. Let us go back to the landscape example, and assume that you are hiking across irregular, natural terrain, such as a forest. You have no specific destination but are just exploring and enjoying the sights and sounds around you. You have a local prospect that includes both free spaces where you can walk and obstacles, such as tree trunks, brambles, or muddy puddles. If you are not careful, you may fall in the mud or become stuck in the brambles. In these circumstances, you will typically be very conscious of every step you take, carefully monitoring the movement of your legs, while looking ahead and considering alternative routes in order to plan your next steps. This is not just conscious reasoning or deliberation: you moreover *feel* the balance of your body with respect to the puddle or how the mud shifts under your foot. This conscious feeling, which includes visual impressions, physical sensations, emotional reactions, and thoughts about the situation, is what is called *subjective experience*.

Imagine now that you are walking along an empty street while having an animated conversation with a friend. This movement happens automatically, following in the footsteps of your friend, without any hesitation or deliberation. However, you still need to repeatedly lift up one leg while putting down the other in the exact spot that allows you to move forward at the right pace without losing your balance. Yet, you are unlikely to feel when and how your foot touches the ground. Your subjective experience will be focused on the conversation, while ignoring the sensations of moving your legs, or the decisions to be made of where to put your foot down next. Here, the process of walking is nearly wholly unconscious. By the time you reach your friend’s house, you may not even remember where you passed or how long you have been walking.

PP theorists are likely to explain the difference between the two situations by noting that walking on irregular, muddy ground is intrinsically less predictable. Therefore, the brain must invest more energy in producing alternative predictions so as to be ready with a better alternative when the initial “active inference” produces a wrong result (e.g., your foot slipping in the mud instead of providing a stable support). Thus, PP tends to explain consciousness as the result of *low precision* in the brain’s ability to predict [15,40].

Nevertheless, you may also walk consciously in situations that can be predicted with high precision; for example, across the smooth, rectangular floor of the exhibition hall of a museum. Here, you amble slowly, being more aware of the sensations you feel in your body and the sights that surround you, while deciding to advance first towards this exhibit, then towards this other one. What characterizes such a conscious activity is that you are deliberately choosing what to do or what to focus on next. This is the aspect of consciousness that is commonly understood as *free will* [2,41].

While free will is often dismissed as a metaphysical concept that escapes scientific investigation, there is a very concrete, pragmatic difference between actions that you freely choose to perform and actions that you perform automatically or involuntarily, outside your will. Imagine that one of the exhibits is a precious vase on a stand. You are aware that if you were to come closer and push the vase from its stand, it would fall and break into pieces. While you are not planning to do this, pushing the vase is part of your prospect of potential developments: you can imagine perfectly well how it would fall if you bumped into it, so you try to move very carefully while examining it.

Suppose now that—after all—you decide to give it a push. Perhaps you want to make a statement against the owner of the vase, or perhaps you are just curious to see how it would shatter into thousands of fragments. Assuming your act of vandalism has been witnessed, you most likely will be brought to court. There, the judge will hold you responsible for the damage you produced, and give you a deserved punishment. That is because the judge will rightly assume that you performed this act out of your own free will—i.e., consciously, deliberately, or voluntarily. This assumption of conscious choice is crucial when making judgements about guilt or responsibility for some action [41].

Now imagine a similar scenario where free will is not involved. Suppose that a wasp has gotten into the sleeve of your coat without you noticing it. While you are standing close to the vase, the wasp stings you in the armpit. The sudden pain makes you violently jerk your arm forward, pushing and thus breaking the vase. This movement is an involuntary reflex, which you could not consciously control. A witness of this event would rightly conclude that the breakage was not deliberate, and therefore that you do not deserve to be punished. Being stung by a wasp was not part of your prospect, so you could not consider different possible reactions. On the other hand, a sudden sharp pain is a condition that your brain instinctively recognizes as a trigger for immediately pulling away from the source of the pain. This is an automatic reaction, leaving no choice in how or whether it is executed.

These two scenarios make clear that there is a fundamental difference between free or conscious decisions, and automatic or unconscious ones. With the conscious ones, your brain somehow maintains a prospect, i.e., a range of conceivable developments to consider. That allows it to choose one option among several. With the unconscious ones, your brain immediately produces a specific response, ignoring any other possibilities. That is because its predictions follow a rigid neural structure, determined by instinct (genes) or conditioning (learning by repeated reinforcement). Let us try to understand the origin of that difference by investigating the neural processes involved in making such decisions.

## 5. Consciousness as a Circulating Neural Process

In our paper introducing LPT [4], we explained the neural mechanisms that could implement conscious deliberation and experience in the brain. Let us here summarize the core ideas. Inspired by the global (neuronal) workspace theory [6,42] and adaptive resonance theory [7], we see consciousness as supported by a process of recurrent or circulating neural activation. The cyclical nature of this process allows it to achieve a state of self-reinforcing *resonance* [43], where the neurons participating in the activation pattern continuously pass activation back and forth, thus maintaining a coherent, synchronized, or integrated activity.

Without this amplifying feedback loop connecting different parts of the pattern, activation would just advance in a straight sequence across successive layers of neurons, ending in a particular “conclusion” or “decision”—e.g., to perform a particular action or to identify a particular perception as a cat. Such a *feedforward* process is quick and efficient, but unconscious [44,45]. The reason is that no activation is left to keep track of the intermediate processing stages, so that they cannot be monitored or redirected by other parts of the brain. That is why you have no idea how your brain came to the conclusion that what you saw was a cat, and not a dog. It also explains why your awareness of the fragility of the vase could not stop your arm from jerking towards the vase when stung by the wasp.

The circulating activation of a resonance, on the other hand, is what maintains the information being processed in *working memory*, so that other parts (“modules”) of the brain have the time to work with it. That means that the content of your consciousness is not just a snapshot of your sensations at this precise instant: it covers a “thick moment” or “extended present”, i.e., a finite temporal interval that includes sensations felt earlier or anticipated to happen shortly [2,46]. The network of neurons in which this activation circulates has been conceived as the *global workspace* of the brain. It functions as a forum through which different, more specialized neural circuits or modules, located in different parts of the brain, can share information [5,6,42].

Since LPT conceives of consciousness as a *process* [4], not as a material component or substance, the theory does not make any claims about where in the brain this “workspace” would be situated. It seems plausible that the resonance would be *distributed*, having neither a specific location in space nor a precise moment in time. It may even extend outside the brain, if we consider the on-going feedback loop between action and perception of the action’s result as part of the circulating process that maintains a coherent activity [47,48,49]. For example, for blind people sensing their surroundings with a cane, their fine-grained control of the cane’s movement and perception of the feedback from the cane’s tip are so tightly integrated with the brain’s prospect that their sensations appear to extend into that tip [50].

In that respect, LPT is compatible with the information integration theory (IIT) of consciousness, which sees a system (neural or other) as conscious if its processes are tightly integrated, so that they cannot be subdivided into independently acting parts [51,52]. However, LPT is more concrete than IIT in specifying the adaptive, goal-directed functions (as summarized by the six aspects of prospect) that this coherent process serves. Therefore, LPT—unlike IIT—would not attribute consciousness to a complex, highly integrated circuit of transistors or other inorganic components that lacks goal-directedness or agency.

Our proposed theory is also compatible with Daniel Dennett’s view of consciousness as *fame in the brain* [53,54]. Both views suggest that neural processes become conscious when they achieve some form of prominence that makes them broadly noticeable throughout the brain. This visibility or “fame” is achieved through the amplification of the pattern of neural activation, temporary maintenance in working memory, and broadcasting to other parts of the brain, thus winning the competition for attention with activation that remains weaker and local.

In conclusion, we see consciousness as an emergent, intense, and coherent *self-maintaining process* [55], with no fixed location, fixed moment in time, or fixed substrate, which however can to some degree retain a continuous identity while undergoing constant change. Physical analogs of such a process can be found in *dissipative structures* [56,57], such as convection cells, tornados, or hurricanes, which consist of a circulating flow of ever-changing material. While moving across a landscape, a tornado can pick up objects, keep them suspended in the air for a while, and then drop them somewhere else. Similarly, the circulating activation that underlies consciousness can pick up information coming from perception, long-term memory, or thought, keep it in working memory for a while, and then drop it again, in order to make room for new information. Such a relatively persistent but adaptive process can perhaps be modeled by means of self-maintaining reaction networks known as *chemical organizations* [58,59].

## 6. Subjective Experience

Global workspace or “fame in the brain” theories provide plausible models for what is called *access consciousness* [2,60]. That means that information held in consciousness (workspace) can be accessed by other parts of the brain, so that it can be examined, monitored, and reported on (e.g., by speaking about it). However, the supposedly “hard” problem consists of explaining the nature and function of *phenomenal consciousness*, or what we called *subjective experience*. This refers to the feelings, sensations, and potentials that open up in one’s field of awareness upon generation of a conscious thought or perception.

**Figure 1:**
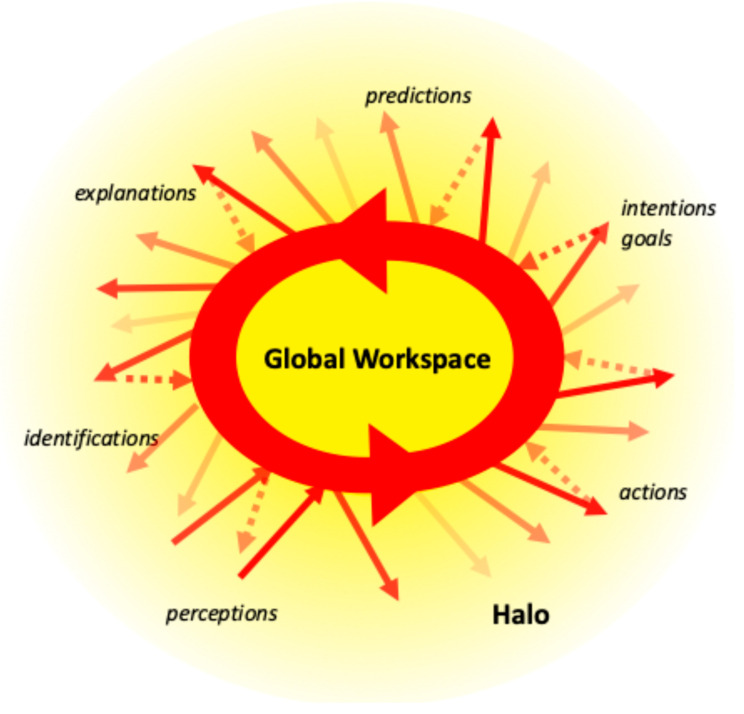
Consciousness as a process of circulating activation. The thick red arrow represents activation circulating in the global workspace, constituting the core resonance. Thin red arrows represent activation spreading into the surrounding halo of associated neural regions. Dotted arrows represent feedbacks; darker colors correspond to more intense activation. The spreading arrows define a prospect of more or less likely potential developments of the core, with intensity proportional to the probability or desirability of the development.

LPT extends global workspace theory by considering a *halo* of weaker, diffuse activation emanating from the core of circulating activation [3,4]. This halo is not itself part of the resonance, but some of it may feed back into this circulating flow, thus guiding its further evolution. (In the tornado analogy, the equivalent of the halo would be the surrounding regions of high and low pressure that direct the winds in and out of the tornado itself.) This halo can now implement the local prospect associated with the percepts or concepts held in access consciousness/global workspace. It provides a prospect in the sense that it “illuminates” or pre-activates a space of more or less probable next developments of the core resonance (see Figure 1). The activation *spreading out* of the resonance is weaker than the self-reinforcing activation *circulating within* the resonance. Nevertheless, it primes or facilitates the extension of the resonance to these already partially activated regions. Thus, the contents of the halo constitute a kind of fringe between conscious and unconscious [61,62]: outside the focus of attention, but still able to affect the evolution of this focus.

The shape and extent of this halo depends on the mechanism of *spreading activation* [63]: the stronger the associations between a region inside the resonance and one outside the resonance, the more activation the latter region will receive. Thus, percepts or concepts that are strongly associated with what is presently in the resonance are more likely to become part of the resonance in the next step. The strength of these associations depends on the amount of reinforcement they have received while the agent was learning from its environment. The content and relative importance of the associations can be understood through the six aspects of prospect mentioned earlier: the brain will pay particular attention to associated perceptions, identifications (concepts or categories), explanations, predictions, intentions (goals), and actions (affordances).

The feelings that constitute subjective experience will in particular include the subjective value (positive or negative) or *valence* of potential developments [64] associated with the concepts presently held in mind. Thus, the halo is not an objective analysis of the content of consciousness, but a subjective evaluation of the aspects relevant to the individual undergoing the experience. It situates this content in a semantic field of relations, expectations, and values, which forms a subjective context. That gives the content meaning, while creating a prospect of likely or desirable future developments. These function like *forces*, pushing and pulling the focus of thought in different directions, typically towards desirable or important options and away from undesirable or indifferent ones. That is why subjective experience is *affective*: it consists of feelings that affect the mental process, creating a complex dynamic whose result is difficult to predict.

This complex dependency between the thoughts held in working memory (global workspace) and the halo of “forces” mapping out a prospect of potential further thoughts or actions provides an answer to a classic philosophical question: why are our thoughts and perceptions accompanied by feelings? Why do we not just automatically process the information received in order to determine the correct reaction and then execute that action? The latter is what a mechanical system, such as a computer or a robot, would do. It is probably also how a simple organism without full consciousness, such as a bacterium or an insect, functions.

The difference can be illustrated by an event one of us (FH) witnessed. A colleague tried to get rid of an annoying wasp by capturing it under an upside-down glass. However, the edge of the glass came down on the wasp’s narrow waist in such a way as to cut off the rear half of its body. To our surprise, this did not kill or even immobilize the wasp: the front half, including the head and first pair of legs, for several minutes continued crawling around and trying to fly off as if nothing had happened. A plausible interpretation is that the wasp’s genetically programmed condition–action rules had not prepared it for a condition in which it lost half of its body. Therefore, its automatic reflexes controlling the movement of the front legs and wings continued executing the same actions as before.

It is clear that the pain felt by a human in a comparable situation of, say, losing one’s legs, would be such that continuing ordinary actions would be the last thing on that person’s mind. According to LPT, that is because pain or other feelings function to shape a prospect of potential developments, making certain developments more probable or desirable, and others less so. Thus, feelings alert you to the need to *consider* appropriate actions, rather than immediately reacting by applying an automatic condition–action rule [4]. Imagine that most of your body became covered under falling rocks. The pain would tell you which parts of your body are most severely hurt or most under pressure, and which ones you might still be able to move so as to possibly free yourself from under the rock without causing further damage. Freeing yourself from under a heap of rock is an activity that requires full consciousness: the risks of getting stuck or getting irreparably hurt are too great to rely on any automatic reflexes; you need to carefully sense and map out the landscape of possible movements and their likely consequences before deciding which limbs to move in what way.

This example illustrates the crucial role of feeling in shaping the local prospect. This applies not only to intense feelings such as pain, but to a great variety of subjective experiences and impressions, such as warmth, touch, taste, shape, and sound, which together color the landscape of potential developments as it appears before consciousness—i.e., the circulating process in the global workspace. That helps consciousness to make a wise, considered choice out of the prospected options, instead of directly executing an automatic condition–action rule or stimulus–response procedure—the way a wasp would do. If there were just a single, easily computable, optimal response to a given condition—the way PP and many cognitive models assume—then there would be no need for feeling to finely appreciate, discriminate, and evaluate the different options for thought or action. Feeling only makes sense in the “extended moment”, the time interval between stimulus and response during which the circulating process can explore a range of potential reactions.

## 7. Decision-Making in the Workspace

We still need to explain how a particular thought, interpretation, or action is selected from within the broad prospect. The global workspace theory of consciousness is inspired by what in AI is known as a *blackboard architecture* [65] and the associated notion of the *society of mind* [66]. The idea is that the intelligence of the mind is actually the *collective intelligence* emerging from the collaboration between an assembly of simple modules, each of which is specialized in a particular type of task. While these modules are individually very limited in their capabilities, together they can solve complex problems, by each taking on the subproblems that they are most skilled in.

The modules communicate by using a shared “blackboard”, “message board” [30], or workspace, in which they post their provisional conclusions. These incomplete results are then picked up by further modules accessing the workspace and adding their own contribution, so as to develop the result a little further. By thus building on each other’s results, the “society” of modules can tackle complex problems with many aspects. This mechanism is similar to the coordination via stigmergy that social insects, such as ants and termites, use to make collective decisions and to develop complex structures, such as a termite hill or a network of pheromone trails [67,68].

While activity performed by independent agents can be efficiently coordinated by having those agents work on a shared medium—such as a blackboard, pheromone trace, or neuronal workspace—there is an intrinsic *indeterminism* in how this activity proceeds. In the simplest case, each agent executes a specific condition–action rule: whenever the content of the medium satisfies a condition it recognizes, the agent performs the associated action, thus changing the content of the medium—a change perceivable by the other agents. This changed content will typically be recognized as satisfying conditions that incite one or more agents to perform a subsequent action. In this way, actions build on the outcomes of previous actions [68].

The problem is that, in general, the state of the medium will simultaneously satisfy the conditions recognized by different agents. In this case, it is not clear which of the associated actions should be performed first. Depending on the order in which actions are executed, the further development of the medium can follow very different trajectories. Such a process is *sensitive to initial conditions* [69]; depending on which action producing a new condition is performed first, the subsequent course of action can go in completely different directions. That is because each next action reacts to the most recent outcome of the sequence of all preceding actions.

We may assume that the propagation of activation in the brain happens *asynchronously*; there is no master clock that times at exactly which millisecond each neuron is supposed to fire. Therefore, there is no fixed order in which neural signals arrive in a particular region, such as the one functioning as a global workspace. Even if we would assume that each module, agent, or condition–action rule in the society of mind would react deterministically to the input it receives, their outputs would arrive in the workspace in a variable, indeterminate order. Because of the non-linearity and path-dependence of the overall dynamics, such small variations in timing can produce large variations in the eventual sequence of decisions made in the workspace [70]. That would make the process as a whole unpredictable. (Note that the neural activity that constitutes the core consciousness, i.e., the resonance, is generally assumed to have become synchronized by participating in that resonance [43]. However, that synchronization does not extend to neural inputs originating in the halo, i.e., outside the resonance.)

A lack of synchronization between neural reactions originating in different parts of the brain is just one of the mechanisms that make the overall process unpredictable. Other sources of stochasticity can be found at the cellular level of neurons and synapses, or even at the microscopic level of the molecules reacting inside these cellular structures [71]. However, there is no need to go down to the quantum level of individual molecules to explain that conscious decisions are intrinsically unpredictable [72]; the observation that the brain obeys a non-linear, and typically chaotic, dynamics [73] is sufficient for that.

This indeterminism explains why the path across the prospect that is eventually chosen in the global workspace is *inherently uncertain*. It also explains why a metaphysical assumption of determinism at the level of local physical processes—which is anyway in contradiction with quantum indeterminacy—does not preclude a pragmatic assumption of free will at the level of the global decision. Thus, we might say that the uncertainty of the choice made derives from the distributed character of the overall process; in the brain, there is no central location with a central time where conscious decisions are made [2]. Decisions emerge from a multiplicity of local processes, initiated at different locations and at different times, which interact and combine into the global resonance that dominates activity in the global workspace. This is a highly non-linear process of self-organization, in which initially independent local processes try to achieve a global alignment [55,74]. Depending on the precise strength and timing of these inputs, the resonance may shift into very different directions.

While this intrinsic uncertainty of the outcome is easy to demonstrate with mathematical or computational models [70], it can also be understood via a more intuitive analogy [4]; it is as if the different modules forming the society of mind are holding a conversation in the forum provided by the global workspace. Depending on which one speaks first or reacts first to a previous proposal, the discussion can veer into different directions, coming to very different conclusions [75].

As an aside, it is worth noting that the common assumption that physical processes are deterministic is metaphysical—not scientific. Some scientific theories—such as classical mechanics or general relativity theory—assume that the outcomes of processes are completely determined by their initial conditions. Other theories—such as quantum mechanics, statistical mechanics, or Darwinian evolution—do not make that assumption. Thus, determinism is a property of a particular theory, not of the underlying reality. There is no experiment that could be performed to test if reality is deterministic. Whether the result of the experiment was accurately predicted or not, determinists will assume that the result was anyway determined before the experiment took place, while indeterminists are free to assume that a different result was in principle possible. There is no way to prove or falsify either assumption. Therefore, the metaphysical assumption of determinism is irrelevant to the practical question whether a decision was made according to a preprogrammed automatism or a result of free will—here understood as the outcome of a discussion between modules. The only pragmatically relevant question is whether the decision is predictable or not. We just argued that in the case of decisions made in the global workspace, we intrinsically *cannot* make reliable predictions. Therefore, our theory assumes uncertainty in the outcome of the decision.

## 8. Towards a Mathematical Model of Prospect

**Figure 2:**
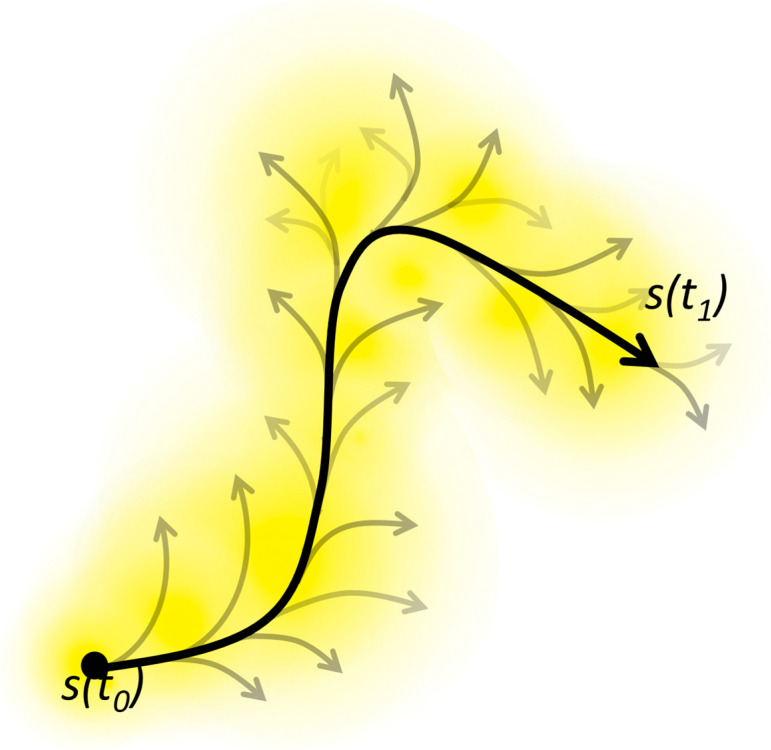
A trajectory through an evolving prospect, from the initial “here-and-now” state *s*(*t*_0_) to the later state *s*(*t*_1_ > *t*_0_). The intensity of the yellow gradient represents the probability density *P*(*s*,*t*) of potential states *s* within the state space *S*. The extent of the yellow zone represents the (local) extent of the agent’s prospect. Arrows represent potential developments within the prospect. The thick black arrow represents the trajectory eventually realized, while the gray arrows represent trajectories considered, but not realized. The subsequent yellow zones along the trajectory represent how the prospect shifts as time advances, similarly to a wave of probability density that propagates across the state space.

Now that we have established that the trajectory followed by a conscious process is inherently uncertain, we wish to more formally examine how this uncertainty is reflected in the local prospect. We defined a prospect as an incomplete, and increasingly fuzzy, outlook, centered on the here and now of the agent’s understanding of its situation *s*(*t*_0_), on a space of potential developments, *S* = {*s*}, departing from that situation. The situation or state *s(t)* can be defined as the whole of the conditions that the agent has inferred or perceived to be present at this moment *t*—albeit that these inferences can be uncertain. A development is a sequence of more or less probable future conditions that the agent’s condition–action rules can infer or achieve starting from these present conditions. Each conceivable development corresponds to a path in that space, while each point in the space is a potential destination through which one or more paths may pass (see Figure 2).

A prospect could be represented mathematically by an analog of the wave function of quantum mechanics. That wave function would be spread out across the space, having different intensities at different points, with the highest intensity typically concentrated in the here and now—i.e., the most recently perceived and identified conditions. In quantum mechanics, the probability density of finding a particle in a particular point is given by the square of the modulus of the (complex-valued) intensity of the wave function at that point. In modeling a prospect, we have no particular reason to work with complex-valued functions. The most straightforward model would be a prospect function *p*(*s*,*t*) whose value simply represents the probability *P*(*s*,*t*) of being in or reaching the point *s* in the prospect at time *t* (Figure 2).

That would allow us to calculate the overall uncertainty of the prospect using the standard formula for entropy *H*, for, respectively, a discrete and a continuous prospect:

$$H\left(t\right)=-\sum_{s}P\left(s,t\right).\log P\left(s,t\right)$$

$$H\left(t\right)=-\int P\left(s,t\right).\log P\left(s,t\right)ds$$

Note that while a prospect is inherently uncertain, it is of course so to a limited degree; the prospect represents the expectation that the agent has about the most likely implications of the situation as it perceives and understands it, excluding unlikely or impossible developments. Thus, a prospect expresses the *meaning, sense*, or *interpretation* that the agent has made about its situation. As such, a prospect carries semantic and pragmatic information *I*(*t*) about the situation. This fits in with the *information integration theory* of consciousness [51], which sees a quale (the content of consciousness at a particular moment) as a cluster of integrated information. The amount of information in a prospect can be defined by another standard formula (where *H_max_* represents the entropy of a perfectly homogeneous probability distribution, which considers all potential developments as equally likely):

$$I\left(t\right)=H_{max}\left(t\right)-H\left(t\right)$$

This is the simplest model of prospect, in terms of a probability density. However, we noted that the space of potential developments can also be conceived as having multiple dimensions, including at least the six aspects discussed earlier. Including these in the model would turn the prospect function into a multidimensional vector **p**(*s*), rather than a scalar *p*(*s*). Such a prospect would be more similar to a force field than to a wave function or probability density. That is why we initially thought of local prospect as a local field [4]. The field lines associated with each point *s* might then be interpreted as the trajectories pointing towards the most likely, important, or desirable next states (Figure 2). That would make the field similar to the phase portrait of a dynamic system, with the attractors playing the role of goal states to which the system’s actions make it converge [22]. However, modeling an individual’s cognitive and physical actions as a deterministic dynamic system contradicts the assumption of uncertainty that is the foundation of LPT. Therefore, it seems simpler at this stage to stick with a scalar prospect function, allowing a straightforward interpretation in terms of probability.

When modeling the six aspects of prospect, probability would be the natural way to measure the prediction component (how likely is the prediction to come true?), but also the related components of perception, identification, and explanation (how likely is the perception, identification, or explanation to be correct?). PP assumes that the brain uses Bayesian algorithms to calculate the relevant probabilities. On the other hand, the evaluation and action components, which express the preferences or choices to be made by the agent, are traditionally modeled by a means of a *utility function*, i.e., a measure of subjective value. A standard decision-making model would then evaluate options according to their *expected utility*, i.e., the utility of the outcome multiplied by the probability that the outcome would be realized. That would require a prospect function with two dimensions, probability and utility, making the calculation of uncertainty unclear.

In standard models of decision-making, this is no problem, because the individual is assumed to be optimizing, i.e., choosing the option with the highest expected utility. However, the prospect interpretation of consciousness assumes that making a choice for a certain interpretation or action merely shifts the prospect to a new local situation *s*. That means that it opens a new range of possibilities locally reachable from that new position, so that uncertainty remains approximately constant. This is similar to the *collapse of the wave function* in quantum mechanics, where an observation reduces the uncertainty of the variable that was observed (e.g., the position *q* of a particle), thus “collapsing” the probability distribution to the value that was measured [76,77]. Nevertheless, the quantum uncertainty principle states that this reduction in uncertainty in one variable must be accompanied by an increase in uncertainty in a complementary variable (e.g., the momentum *p* of the particle), so that the total uncertainty cannot decrease below a minimum value specified by the famous Heisenberg inequality:

$$∆q\cdot ∆p\geq \frac{h}{4\pi }$$

LPT does not have a notion of complementary variables and does not assume a minimum value for uncertainty. In principle, a prospect with zero uncertainty, meaning that only a single development is considered for realization, would define a wholly unconscious activity. This can be conceived as a purely automatic or robotic behavior, as exemplified perhaps by sleepwalking or the arm jerking away from the wasp sting. Therefore, we may assume that the uncertainty of a prospect can take on a wide variety of values, and that higher values indicate more conscious states, in the sense that the mind is considering a wider range of potential developments. However, to do that we still need to define a scalar prospect function.

As first suggested, the simplest way to model the overall evolution of uncertainty during consciousness is to reduce all dimensions of prospect to probabilities. This is what the active inference assumption in PP does, reducing goals and intended actions to predictions that a certain action will be performed, or that a certain goal will be achieved. That allows us to replace the “expected utility” of a goal or action with the probability that the agent would effectively select that goal or action for execution.

In our cybernetic interpretation of agency, the elementary components of prospect are not predictions, but condition–action rules, with actual predictions interpreted as internal, cognitive actions of inferring a subsequent condition. From that perspective, each condition–action rule has a certain probability to be executed in a given situation, i.e., a conjunction of conditions. That would allow us to model prospect as a probability distribution over a space of condition–action rules. A plausible constraint on that distribution is the assumption that rules whose conditions do not match the present situation have zero probability. At each moment, one or more of these rules would then be selected with the corresponding probability and executed. The corresponding action(s) would produce one or more new conditions, which would in turn induce a new probability distribution over the applicable condition–action rules, and thus a new prospect.

## 9. Degrees of Consciousness

Having defined consciousness as a prospect of potential developments characterized by the uncertainty *H*(*s*, *t*), we can now classify different states of consciousness by their degree of uncertainty. As noted, prospects with zero uncertainty, *H*(*s*, *t*) = 0, then correspond to unconscious states, in which the brain functions on automatic pilot, always selecting the most probable next state, and never taking the time to consider alternatives.

Given that we do not know how large the space *S* = {*s*} of potential developments (modeled as condition–action rules) is, we cannot calculate the upper bound *H_max_* on the uncertainty function. Even if we could define a bounded subspace *S*, it seems extremely unlikely that the probability distribution over this subspace would ever approach homogeneity. Such a maximum entropy *H_max_* would mean that the neural networks of the brain are absolutely indifferent as to which development they consider more likely. That would imply that the agent has no clue about what to expect or what to do next; it would have no information whatsoever about the situation: *I* = 0. Perhaps an analog of such a state could be found in states of extreme drunkenness or psychedelics-induced delirium, characterized by complete disorientation. This is obviously not a healthy state to remain in. However, milder degrees of high entropy states may have therapeutic applications.

Some theories of the effect of psychedelic drugs posit that these drugs increase the entropy in the brain [78,79], reducing the constraints that otherwise canalize neural activation into following well-worn paths. From the PP perspective, this means that psychedelics relax the precision weighting of predictions deriving from prior beliefs about how the world is structured, thus allowing lower probability perceptions and interpretations to come to the fore and to be consciously considered [80]. This “loosening” of cognitive constraints may allow people to escape from rigidified patterns of thinking and feeling, freeing up bottled energy, and inspiring novel perspectives and creative ideas [81]. That is why psychedelic drugs in the form of microdosing or supervised sessions are increasingly being used in psychotherapy to help people break out of obsessive thinking or emotional and cognitive imprints of traumatic experiences [2,80], which are characterized by a narrow focus on particular problems and a concomitant neglect of perceptions, feelings, and actions outside that focus.

However, there are more natural and less risky methods to broaden the focus and thus increase the prospect and its associated uncertainty *H*. The *broaden-and-build theory* of positive emotions [82] proposes that positive feelings, such as relaxation, joy, or amusement, function to broaden our field of awareness. This broadening leads us to consider a much wider range of sensations, thoughts, and actions. That helps us to acquire a greater variety of experiences, insights, and social connections, on which we can then build a better life, a greater resilience, and a greater ability to cope with challenges. Negative emotions, such as fear, anger, or shame, on the other hand, make us focus on the problem that caused the emotion, so that we gather the energy to combat the problem, or at least make sure it does not get out of hand.

The corresponding difference in prospect can be illustrated with two scenarios. In the positive emotion scenario, a tourist on vacation is sauntering through a beautiful city, enjoying all the sights and sounds, occasionally stopping to try some food, buy a souvenir, or chat with the locals. In the negative emotion scenario, a businessperson who is late for an important meeting is rushing through that same city, looking out only for potential obstacles on the shortest path to the destination, while ignoring everything else.

**Figure 3:**
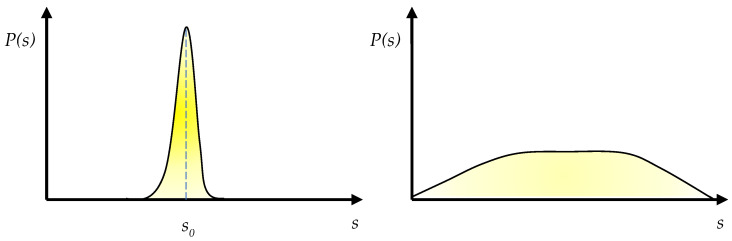
Two probability distributions *P*(*s*). Left: representing a narrow, low-entropy prospect centered on the state *s*_0_; right: representing a broad prospect with a high entropy.

We can visualize the corresponding probability distributions *P*(*s*) as the curves depicted in Figure 3. In the case of the harried businessperson, the curve is tall and narrow, peaking on the shortest path *s*_0_, with everything that does not interfere with that path having a near-zero probability of being considered. In the case of the relaxed tourist, the curve is so flat that there is not really any central peak, because different directions for walking or looking appear equally interesting, and the probability distribution only starts to diminish for ventures far from the center that appear less attractive.

There are other mechanisms next to psychedelics and positive emotions that can broaden the prospect and thus increase the entropy *H* of the probability distribution. *Mindfulness* is a state in which one maintains a broad, open awareness, allowing a wide variety of sensations, feelings, and thoughts to be experienced, without fixation on any single one of them [83,84]. In this state, awareness is inclusive and fluid, allowing a person to notice thoughts, emotions, and sensory inputs as they arise and pass. The key to achieving mindfulness is to be non-judgmental. That means that you do not judge or evaluate phenomena that appear in consciousness to be good or bad, important or insignificant. That allows at first sight minor or unpleasant stimuli to still enter consciousness, without being immediately suppressed by the dominant thinking patterns.

The state of mindfulness has been shown to provide a range of health benefits, including reduction in anxiety, stress, and depression; better emotional regulation; greater cognitive flexibility and attentional control; reduction in pain and sleeplessness; and lower blood pressure and heart rate [85,86,87]. While these benefits have been confirmed by Western medicine and psychology, the concept and practice of mindfulness originate in Buddhism. It is worth exploring the reasoning behind this ancient philosophy, which—when making abstraction of certain metaphysical assumptions such as the eternal cycle of reincarnation—is remarkably consonant with contemporary neuroscience in its understanding of consciousness [2,9].

## 10. Conditioning and Breadth of Prospect

According to Buddhism, the mind is conditioned by different “attachments”. These are desires or cravings that pull us towards certain objects or conditions, and aversions or fears that push us away from other objects or conditions. An example of a craving is the one experienced by a smoker for a cigarette when seeing other people smoke. An example of an aversion is the panic some people feel when asked to speak before an audience. In these examples, the desire or fear strongly *determines* or *conditions* the behavior of the person. The smoker will find it very difficult to resist the temptation of lighting a cigarette that is offered, while the person with public speaking anxiety will find it very difficult to accept a speaking invitation. That means that they are nearly certain to execute the following *condition–action rules*:

Smoker: being offered a cigarette (*condition*) → smoke (*action*)
Speaking anxiety: being asked to address an audience (*condition*) → refuse (*action*)

If we label the condition as *c* and the action as *a*_0_, then the *conditional probability* of the action given the condition, *P(a*_0_|*c*), would be nearly 1. That also means that the probability of executing any alternative action *a_i_* (*i* ≠ 0) would be nearly 0. In other words, the entropy of the probability distribution of potential actions given the condition would be nearly zero. The condition in this example strongly determines the expected action. Other conditions are likely to be less constraining in the way the subject reacts to them.

Nevertheless, Buddhism assumes that we are all rather strongly conditioned to react in habitual ways to our different “attachments” (desires and fears). These conditionings result from a lifetime of positive and negative reinforcements, mostly coming from the reactions of parents, peers, and society, which imposed certain behaviors or ways of thinking and suppressed others. “Karma” is the term Buddhism uses to refer to our habitual attitudes, aversions, intentions, and actions that result from and add to this accumulated burden of conditionings. It denotes both the action or cause and the results of that action, which may manifest as an attitude that leads to further unpleasant experiences. (Karma can also be understood as a potential for outcomes—an interim phase between the intentional action that serves as the cause and its eventual results.)

The problem is that many of these rigidified attitudes are inadequate, making us desire things that do not bring real satisfaction (such as cigarettes, wealth, or fame) and fear things that are not actually harmful (such as public speaking, criticism, or being ignored). This continuing chase after things without genuine value and worry about things that are not genuinely important results in an unpleasant state called “dukkha”. This term is commonly translated as “suffering”, but perhaps more adequately as “unsatisfactoriness” [9]. It can be understood as an inability to achieve true happiness and peace of mind. Fully liberating oneself from such attachments would bring about a kind of awakening or enlightenment, which is, depending on the tradition, known as “nirvana”, “nibbana”, or “satori”. This is a state of detachment, deep insight in the functioning of one’s mind, and general wisdom. It results in a genuine freedom to either ignore or follow through on the conditions we experience, instead of automatically reacting in our habitual manner (like becoming anxious at the thought of public speaking).

Buddhism has developed different methods to reduce the strength of the conditionings that pull our attention towards or away from specific objects of attachment, thus broadening the range of consciousness, and removing the “root causes of ignorance”. We already discussed mindfulness meditation as an open, non-judgmental attitude that allows minor sensations to enter consciousness without attempting to remove or resist them. A method complementary to such *open monitoring of experience* is *focused-attention meditation* [83,88], sustaining conscious focus on a single item, such as your breathing, a word or concept, or an object, while resisting distractions that attract attention to other things.

This is intrinsically very difficult because the focus of consciousness tends to be shifting continuously, engaging with either thoughts about issues we consider important (e.g., ruminating about how we would deal with a public speech) or with different perceptual stimuli (e.g., a noise, or something moving). This restlessness and constant jumping from topic to topic is known in Buddhism as the “monkey mind”. Meditation is a method intended to train consciousness to ignore such distractions that pull attention away from the focus. This is not achieved by *suppressing* distracting thoughts, which is impossible (as illustrated by the injunction “do not think about a white bear” [89]), but by not engaging in them, while systematically returning to the original focus. That allows experienced meditators, such as Tibetan monks, to achieve seemingly extraordinary capabilities, such as ignoring strong cold, pain, or hunger [90,91,92].

This general ability to not react in an automatic, habitual way to experienced conditions can again be modeled by means of an entropy measure. If we represent the set of potentially experienced conditions as *C* = {*c_j_*} and the set of potential actions in response to these conditions as *A* = {*a_i_*}, then we can define the *conditional entropy H* of *A* given *C*:

$$H\left(A|C\right)=-\sum_{j}P\left(c_{j}\right)\sum_{i}P(a_{i}|c_{j}).\log P(a_{i}|c_{j})$$

Busseniers [93,94] has proposed a measure of the determination of a set *A* by a set *C*, defined by the following formula:

$$Det\left(A|C\right)=\frac{H\left(A\right)-H\left(A|C\right)}{H\left(A\right)}$$

Applied to condition–action rules, this formula quantifies the degree to which actions *A* (which as before include mental actions such as thoughts or feelings) are determined by conditions *C*. If actions were independent of conditions, then the conditional entropy of actions depending on the conditions *H*(*A*|*C*) would be equal to the entropy of the actions *H*(*A*), which does not take into account conditions. In that case, *Det* (*A*|*C*) = 0. That would describe an agent that is completely indifferent to the conditions which it experiences. It is free to do, think, or feel whatever it pleases, independently of its actual situation. This is of course an exceptional state that perhaps may only be temporarily achieved during certain psychedelic or mystical experiences in which the subject altogether loses the sense of being an individual self, subjected to the conditions here and now. However, this is not a state that can normally be maintained over a long term, because it implies that the subject would be completely unreactive to conditions such as hunger, pain, or freezing. According to some interpretations, this is how the historical Buddha, Siddhartha Gautama, died—bringing himself via meditation into a state of nirvana where he no longer experienced the difference between life and death.

The opposite of this state would be one of maximum determination:

$$Det\left(A|C\right)=1$$

Here, there is no freedom, uncertainty, or choice about how to act or what to think: *H*(*A*|*C*) = 0. For every condition *c_i_* that can be experienced, there is only a single possible reaction *a_j_*: *P*(*a_j_*|*c_i_*) = 1, *P*(*a_k_*, *k* ≠ *j*|*c_i_*) = 0. That would describe an agent whose cognitive processes and behavior would be completely automatic or deterministic. That would imply an agent—such as a wasp or a person who is sleepwalking—that lacks any form of consciousness or free will. (Note that we here ignore the possible effects of noise in the wasp’s nervous system that could produce a non-zero entropy in its reactions, because such noise does not define a prospect of potential developments that can be consciously considered.)

The more realistic cases of human consciousness are intermediate between these two extremes:

$$0<Det\left(A|C\right)\leq 1$$

Let us define breadth *B* of prospect (0 ≤ *B* < ∞) as:

$$B=\frac{1}{Det\left(A|C\right)}-1=\frac{H\left(A|C\right)}{H\left(A\right)-H\left(A|C\right)}$$

*B* can be interpreted as a measure of the degree to which consciousness is wide open (large *B*), like in mindfulness or the example of the sauntering tourist, or limited to a narrow range of options (small *B*), as in the example of the harried businessperson (Figure 3). This measure in principle allows us to rank different degrees of consciousness, and thus to distinguish different mental states such as those, e.g., accompanying positive vs. negative emotions, or psychedelic and meditative experiences.

The psychiatrist Deikman has proposed that meditation—whether mindfulness-based or focused-attention—produces a *de-automatization* of patterns of thinking and feeling [95,96,97]. De-automatization is a process that temporarily suspends the automatic cognitive mechanisms of interpretation and reaction to the conditions we sense. That allows for a more direct and unmediated experience of sensory information.

As we noted, much of human perception and cognition operates on autopilot, shaped by ingrained neural connections and conditioned habits. These automatic processes filter, classify, and interpret sensory input based on prior experiences and learned frameworks. Such automatic functioning is efficient and dependable for routine behavior. However, it limits awareness to stereotypical ways of perceiving, understanding, and thinking. These tend to subdivide phenomena into rigid categories [98], such as desirable or aversive conditions, conventional types of objects, and the subject, “I” or self, that perceives these objects. Deikman argued that meditation interrupts these automatic processes, allowing for a less rigidly structured experience of the situation. This hypothesis was empirically confirmed by later research [97,99].

De-automatization allows individuals to experience reality in a broader, more open, and more fluid manner. Such fluid experience includes a more direct awareness of sensory phenomena without labels or categories [98]. It may even lead to a dissolution of the conventional boundaries between the self and external objects—a state of awareness known in Buddhism as *non-duality* or *non-conceptual oneness* [100]—and a heightened sense of unity or interconnectedness with the world. Similar feelings are expressed in descriptions of psychedelic [80], self-transcendent, or mystical experiences in different spiritual traditions [101]. De-automatization through meditation also aligns with the Buddhist goal of liberation from attachments, resulting in cessation of suffering, while increasing creativity and flexibility of thought flow. In our framework, it can be seen as a mechanism for increasing the breadth of prospect *B*, by reducing the strength of conditioning *Det*(*A*|*C*).

## 11. The Experience of Non-Duality

Let us further explore the advanced meditative experiences that can offer glimpses into the nature of consciousness beyond habitual reactivity to external conditions [2,9,100]. This state of awareness can sometimes be achieved by prolonged meditation sessions in which sensory stimulation is reduced to a minimum, e.g., by meditating for days in a dark and silent room or by locking one’s field of vision on a specific spot. That helps the meditator to arrive at a sense of consciousness as an autonomous process independent of external conditions. The following describes how one of us (SB) experienced such a state during a seven-day dark retreat.

Free from predictions, implications, and identifications, yet aware of its condition, non-duality means ceaselessly abiding in the state of full identification with awareness itself. In this state, awareness is all that exists, and the structures previously erected within the consciousness field lose their grounding, which was previously sustained by external stimuli. The dissolution of these structures releases distortions in the consciousness field caused by the constriction of the field to support concepts, such as one’s perceived identity. The dropping of identifications relaxes locked up awareness, allowing everything to return to the process of consciousness. Consciousness is thus restored to its pristine state, free from the social, cultural, psychological, and conditioned identifications assumed falsely as one’s identity. This realization leads to the further understanding that the self as a frame of reference for one’s sense of existence does not truly exist as an independent entity [2,102], and that the belief that one is something or someone—a construction to be maintained through exertion of mental energy—is fundamentally erroneous and burdensome.

This fundamental error is a shared human misconception, a common collective social theater that leads to the persistence of suffering and ignorance and the continuation of the cycle of karma. Therefore, one’s subjective experience is not genuinely novel until this realization occurs. True subjective experience is an inherent property of the consciousness process itself; that is, it is aware of itself. This understanding reveals that the consciousness process is, by nature, dynamic, self-referential, and thus experiential.

This abstract idea can be visualized with the metaphor of a trampoline’s surface. Without any external force, the surface of the trampoline is naturally bouncy. This bounciness is what is experienced by consciousness itself, leading to the generation of thoughts. However, when a heavy object is thrown onto the trampoline, it bounces back and deforms the surface. This temporary but visible deformation suggests that the trampoline’s bounce is caused by the external object. This assumption misplaces the origin of subjectivity, situating the nature of consciousness outside itself. Predictive processing follows this path, in which predictions tell the trampoline how to respond to objects or concepts, shaped by one’s habitual attitudes and automatic reactions. This denies the self-sustaining nature of consciousness, which is independent of cues or impact of predictions.

In this view, the locus of subjective experience is located externally, suggesting that what the totality of one’s subjectivity and measure of consciousness is what enters the brain’s predictions. The “hard problem” of subjective or qualitative experience becomes difficult to address because it is viewed through the lens of consciousness as something essentially passive, inert, controlled by the dynamics and structures of the external world.

Yet, consciousness is aware of itself, meaning that it is the driving force behind the experience of nothing but itself. Conceptual oneness or unity here means that the foundation of all phenomena is consciousness being aware of itself. In other words, consciousness is genuinely and subjectively experiencing itself. This is the qualitative nature of consciousness: a dynamic, self-sustaining, liberated awareness in the driver’s seat. Therefore, to examine the genuine nature of subjectivity the practice of meditation asks the practitioners to investigate the following: Who is having these thoughts [2]? Are they predictions of my brain, or is it awareness itself? Once thoroughly investigated, it becomes evident that the thoughts are generated by the self-observing nature of consciousness and not by a separate entity within the field, unimpeded by the restrictions of the self-concept. The notion of ‘I’ ceases to hold once the true nature of consciousness is experienced [2,102,103].

It is like the sun emerging from behind the clouds—the sky is clear once again. Freed from the clouded veil of implications, the true nature of consciousness reveals itself naturally, and nothing else can claim independent existence in the light of this realization. Consciousness alone is reality. In other words, ‘I’ does not independently exist; my awareness exists, my form exists, but the concept of ‘me’ was never an independent entity—and it never will be. This realization is profoundly liberating, as it dissolves the heavy burden of our concept of existence. Awareness of consciousness itself stands alone, requiring no external support.

The form, now filled with self-knowing awareness of consciousness, gradually transforms into a being filled with positive qualities due to the realization of the non-conceptual oneness of reality. Goodness, kindness, love, and compassion then flow effortlessly from this keen realization, reaching out to connect itself with others. Like a small cloud in the sky, it joins another, and then another, without a second thought.

This shift in the quality of subjective experience does not entail renouncing the material dimension of life. On the contrary, it marks the beginning of something unique: freedom of one’s field of consciousness and thus its opening up to experiencing all that there is. Formulated in the terminology of LPT, this is a state of high tolerance for entropy without disintegration of consciousness into disjoint and bounded concepts. It is important to note that such a state does not imply the absence of pain in one’s subjective experience. However, suffering—stemming from attachments to predicted or desired outcomes—ceases, as the free and pristine nature of consciousness is laid bare to the subject.

## 12. Operationalizing Prospect

The previous sections have proposed an approach for formalizing the notion of prospect by means of an entropy-based mathematical measure *B* of breadth of awareness. However, to be able to test the local prospect theory in the real world, we would also need to *operationalize* this notion; that is, develop methods to empirically measure the breadth of prospect for people in different circumstances—e.g., before and after a meditation session. Directly calculating entropy measures does not seem doable, given that we do not know the extent of the prospected action space *A* or the conditional probabilities *P*(*a*|*c*) of some action *a* given a condition *c*. Therefore, we will need to find plausible *indicators* or proxies for the relevant variables.

Empirical tests of the broaden-and-build theory of positive emotions have used two indicators for breadth of awareness [82]: one for actions and one for perceptions. The action measure is most straightforward: give people a sheet of paper and ask them to write down all the actions they might consider doing at this moment. The larger the number of action items they register, the broader their awareness (or what we call “prospect”). Using this measure, the theory’s main prediction was confirmed: people that were made to feel positive emotions wrote down a broader range of potential actions than people made to feel negative emotions [82]. A related operationalization is the one used to measure *divergent thinking* ability [104,105], which is associated with creativity. Here, subjects are asked to produce as many different answers as they can think of within a given time span to an open-ended question, such as “what could you use a brick for?” A greater variety of answers is likely to indicate a broader prospect.

The perceptual indicator is more subtle: subjects are asked to describe a picture that exhibits patterns at two levels of perception, locally and globally [106]. For example, the picture consists of many small T-shaped patterns that together form a big L-shape. Those who refer to the global pattern (L) are considered to have a broader awareness than those who refer only to the local patterns (T) [82]. Another possible testing paradigm for breadth of perception is to show pictures that include both objects in focus and features in the surrounding context or background [107]. Subjects with a broader awareness are likely to notice, remember, or recognize more background features than those with a narrow focus.

A further promising approach is to measure the degree of automaticity of some cognitive or behavioral performance. In psychology, this is commonly achieved by means of the *Stroop task*. Here, subjects are asked to name the color in which a word is printed (e.g., green), but without reading the word itself, which is the name of a different color (e.g., red). Achieving this for a long list of different color words is difficult, because we are trained to read words rather than articulate the color of their ink. Reading the word is an automatic reaction that must be inhibited in order to efficiently perform this task. There is evidence that meditators perform better in this and similar tasks, suggesting that they are less determined in their reactions by their automatisms [97,99].

A different approach to operationalizing prospect may be *perplexity*, which is a measure of the unpredictability of a pattern. As suggested by the example of the sauntering tourist and the harried businessperson, a person with a broader prospect is likely to behave in a less predictable manner, following a less straightforward or obvious trajectory. Perplexity is defined as an exponential function of entropy [108,109]. Calculating it thus also requires knowledge of an *a priori* probability distribution. While we do not know the probabilities within people’s mental prospect, we may be able to develop a predictive model of their behavior via machine learning methods that induce recurrent patterns from observable behavior. For example, a typical pattern of leg movements during a goal-directed walk across a flat surface is quite predictable. A model trained on common walking data should be able to predict the next step of a standard goal-directed walk with a high probability of being correct, implying a low uncertainty/entropy and therefore a low perplexity. However, that same model would find it much more difficult to predict the trajectory of the sauntering tourist or the hiker in the forest.

Similar measures are used in AI to evaluate the performance of LLMs (large language models) that generate text by each time adding a word to the sequence of words that is already there with a probability conditional on the preceding sequence. Here, the perplexity of a particular sentence can be measured in terms of the probabilities *P*(*s_i_*) assigned by the language model to each of the *N* words (*s_i_* | *i* = 1, …, *N*) as they are subsequently generated to produce the text [110], according to the following formula:

$$Perplexity=2^{-\frac{1}{N}\sum_{i=1}^{N}\log P\left(s_{i}\right)}$$

Such a measure will assign low perplexity (=high predictability) to common phrases, such as “The weather forecast says that it will rain tomorrow”, and high perplexity to uncommon, creative expressions, such as “Translucent flags flutter in the breeze of a turquoise ocean”.

LPT assumes that people with a broad prospect hold a wider variety of potential actions in mind and that they are therefore less likely to produce the most probable or most obvious reaction to a given condition (such as the beginning of a sentence to be completed). That suggests that the language and behavior generated in a state of broad prospect would be characterized by higher perplexity. That hypothesis could be tested using LLMs that can calculate the perplexity of text fragments. A typical experiment would be to let two groups of people each individually write a text on a given topic—e.g., a group that has been meditating and a control group that was occupied with some other activity. LPT would then predict that the texts of the first group would have a higher average perplexity than the control group.

Similar experiments could be performed using predictive models of other sequential behaviors, such as walking, moving the body, or scanning a scene with the eyes. In the latter case, LPT would predict that the trajectory of the gaze of a person in broad prospect mode would be less concentrated on the salient objects in focus (such as people, faces, or eyes), while exploring a wider range of features in the scene, thus being less predictable or more entropic.

Using these and related operational measures, it should be possible to distinguish between different degrees of consciousness as defined by LPT, and to determine which initial conditions (such as positive emotions, mindfulness, meditation, or use of psychedelic drugs) tend to affect that degree.

## 13. Conclusions

We have summarized and further elaborated a new theory of consciousness, the local prospect theory (LPT), which attempts to expand on previous theories of consciousness and shed light on the so-called hard problem of consciousness, i.e., the function of subjective experience [4]. Consistent with predictive processing (PP) theories, LPT assumes that the core function of cognition in the brain is to make sense of experienced situations by identifying, explaining, and predicting perceived aspects of the situation, and using these interpretations to select and perform appropriate actions. However, unlike PP, LPT does not assume that the ultimate aim of cognition is to minimize uncertainty. Maximizing the accuracy of predictions and thus minimizing uncertainty, as PP assumes, is essential for immediately recognizing and reacting to a situation. This kind of sense-making and acting happens automatically and unconsciously; we may be conscious of the result but not of the process through which this result was achieved.

In contrast to such *automatic* sense-making, LPT assumes that *conscious* sense-making requires freedom: the ability to consider different possible developments to focus on, think about, or act towards. That means that there is a space *S* = {*s*} between the stimulus (the condition as sensed) and the response (the brain’s interpretation and choice of action). That implies that the agent has a *range* of potential reactions from which it can choose what to consider or what to do. We call that range the *local prospect*. It is a *prospect* because it provides a view of potential developments that allows the agent to some degree to anticipate or plan these developments. It is *local* because the prospect typically does not extend much beyond the here and now of the situation as presently experienced, becoming increasingly vague for more remote developments.

The prospect is not an objective representation of probable futures, but a subjective appreciation pointing towards possibilities that are important, meaningful, or valuable for the agent. Thus, it is to some degree comparable to a fitness landscape, indicating the locally best (fittest) and worst potentialities, or to a field of forces pushing and pulling the agent towards the good and away from the bad. The different aspects of this field of subjective meaning may be captured by what we introduced as the six aspects of sense-making: perception, identification (recognition), explanation, prediction, intention (goals), and action (affordances). The active inference extension to PP implicitly assumes similar functions. The difference is that PP reformulates all these functions as predictions, including the choice of goals and execution of actions, conceived as *active inferences*. LPT, on the other hand, proposes to model them all as *condition–action rules*, considering predictions as internal (mental) actions of inferring an anticipated condition.

The reason PP reduces all mental activities to predictions is its assumption that the brain’s fundamental dynamics is to maximize the accuracy of its predictions, and thus to minimize surprises. LPT, on the other hand, assumes that there is an essential uncertainty about the best way to act: our goals are not preprogrammed predictions, but choices that are typically freely made by considering a range of options that may be better or worse, but none of which is unequivocally the best. Making that choice requires consideration and deliberation, i.e., taking the time to feel out, understand, and evaluate more than one option. That requires keeping some of the options in mind so that different neural processes react to them.

Building on the global (neuronal) workspace theory of consciousness [5,6], we proposed that this is achieved by the brain maintaining a distributed process of circulating activation—a self-reinforcing process that has been called an adaptive resonance [4,7]. This activation pattern functions as a temporary, working memory of the present situation, providing more specialized brain modules with access to this global understanding. The global workspace theory proposes a plausible model of *access consciousness*, the mechanism that gives the agent access to certain cognitive processes, so that it can to some degree examine and control these processes. This conscious control is what underlies our sense of *free will*, the ability to ignore automatic reflex reactions and weigh the pros and cons of different possibilities before deciding how to act.

We explained this deliberation procedure by using the analogy of the *society of mind*: different specialized modules in the brain collectively solve a problem by proposing partial solutions and reacting to proposals of other modules within the internal discussion forum known as the global workspace. Because modules initiate reactions independently and asynchronously, while their “conversation” is non-linear and sensitive to initial conditions, the outcome of this discussion is typically unpredictable. That explains the apparent indeterminism of free will without requiring any metaphysical assumptions.

We further explained the role of feeling, subjective experience, or “phenomenal consciousness” in helping the mind come to a good decision. Feeling can be understood as the field of “forces” pushing and pulling the resonance in different directions, thus shaping the “fitness landscape” of prospect. Prospect itself can be understood as a halo of activation spreading out of—and feeding to some degree back into—the resonance. This “cloud” or “wave” of diffusing activation prepares the way for shifts in the core resonance, yet without determining its dynamics. This complex neural dynamics is very different from the one underlying automatic, unconscious processes, where activation propagates in a straightforward, bottom-up manner from stimulus (condition) to response (action), or, as noted by PP, in a complementary, top-down manner from prediction to interpretation of sensory data—leaving no time or opportunity for other modules to monitor or intervene.

We then considered more abstract models of local prospect as a wave of probability density propagating across a space of states representing potential developments as envisaged by the brain. The inherent uncertainty of prospect can be expressed by the entropy *H* of this probability distribution, while its information content can be expressed by the difference between this entropy and maximum entropy: *I* = *H_max_* − *H*. A more detailed model considers the probability distribution of condition–action pairs. This allowed us to define the *conditional entropy* of actions *A*, given conditions *C*: *H*(*A*|*C*). That in turn allowed us to define a measure of the determination of actions by conditions: *Det*(*A*|*C*).

Using this measure, we were able to reformulate the intuitive ideas about consciousness formulated by Buddhism in a more precise manner [9]. Buddhism assumes that much of our thinking and behavior is conditioned by “attachments”: automatic reactions to certain conditions as being intrinsically desirable (cravings) or aversive (fears). These conditionings make it difficult to lead a life that is truly free or to achieve long-term, meaningful happiness. Buddhism has therefore developed different practices and methodologies to liberate us from such conditionings. The ones that have been scientifically studied in most depth are *mindfulness* and *focused-attention meditation* [88]. Both have been shown to provide a range of (mental) health benefits [85,91,111], while reducing the automatism of categorizations and reactions [97,99]. In the limit, such practices may allow one to reach a state of complete freedom from conditioning that would correspond to *Det*(*A*|*C*) = 0.

While this is not a state that can be sustained over the long term, it may give us a glimpse of undifferentiated, “pure” consciousness, as a process that can self-maintain even without external stimulation. The hard problem of consciousness may actually consist in our difficulty in grasping this kind of “ultimate” subjective experience [103]. It remains hard in part because we tend to erroneously assume that the drivers of consciousness, typically considered as either the “self” or as external phenomena, are situated outside this self-maintaining process.

We further defined the breadth of prospect *B* in terms of conditional entropy, as a measure of the range of thoughts or actions that are potentially considered under different conditions. The smaller *B*, the narrower the prospect, the more automatic or the less conscious the reactions. Various observations suggest that *B* would be larger in brain states characterized by positive emotions, mindfulness, the controlled intake of psychedelic drugs, and after meditation. To test these hypotheses, it would be necessary to operationalize breadth of prospect, i.e., develop empirical indicators for *B* in different states of consciousness. We suggested a number of plausible indicators, including number of actions considered, the width of perceptual focus, the ability to control automatic reactions, and the perplexity (unpredictability) of language or behavior produced.

Using such indicators and mathematical measures, it should be possible to further elaborate and test the proposed local prospect theory of consciousness [3,4]. While this theory is still in an early stage of development, we hope that our arguments will convince researchers to further investigate the ideas proposed here, and thus hopefully contribute to solving the “hard problem of consciousness”.

## Data Availability

There are no additional data.
